# Characterization of Antioxidant Bioactive Compounds and Rheological, Color and Sensory Properties in 3D-Printed Fruit Snacks

**DOI:** 10.3390/foods13111623

**Published:** 2024-05-23

**Authors:** Anica Bebek Markovinović, Tomislav Bosiljkov, Tibor Janči, Marko Kostić, Nebojša Dedović, Ela Lučić, Katarina Bavrka, Branimir Pavlić, Danijela Bursać Kovačević

**Affiliations:** 1 Faculty of Food Technology and Biotechnology, University of Zagreb, Pierottijeva 6, 10000 Zagreb, Croatia; 2 Faculty of Agriculture, University of Novi Sad, Trg Dositeja Obradovića 8, 21102 Novi Sad, Serbia; 3 Faculty of Technology, University of Novi Sad, Blvd. Cara Lazara 1, 21000 Novi Sad, Serbia

**Keywords:** strawberry, strawberry tree fruit, *Arbutus unedo* L., 3D printing, quality

## Abstract

The influence of wheat starch (6%, 8% and 10%, *w*/*w*) and a 3D printing program (program 1 vs. program 2) on the content of bioactive compounds, antioxidant capacity, color parameters and rheological and sensory properties was investigated in 3D strawberry and strawberry tree fruit snacks. Increasing the starch content led to a decrease in the content of almost all the bioactive compounds, while it had no effect on the antioxidant capacity. The printing program had no significant effect on the bioactive compounds (except hydroxycinnamic acids), antioxidant capacity and color parameters. A higher starch content improved the strength of the sample but had no effect on the mechanical properties. Smaller particles with a higher starch content improved the stability of the sample. In contrast to the programs, varying the starch content had a significant effect on all the color parameters except the a* values. Eight different sweeteners in two different concentrations were used for the sensory evaluation of the 3D-printed snacks. The variations in sweetener content only affected the sweet and harmonious taste. In summary, this study confirms the great potential of fruit bases for the production of 3D-printed snacks with excellent biological and rheological properties, which can be a step toward personalized food with the addition of sweeteners.

## 1. Introduction

Due to growing consumer awareness and increased interest in nutritionally valuable foods, novel plant materials that could improve the nutritional, functional or sensory properties of foods are in high demand [1]. Of particular importance are plants that have not yet been sufficiently researched but show great potential for processing, such as the Mediterranean plant strawberry tree fruit (*Arbutus unedo* L.), which has been shown to have strong biological effects thanks to its antioxidant bioactive compounds [2]. It has already been shown that phytochemicals from *A. unedo* have the ability to slow down the oxidative process by inhibiting the harmful effects of free radicals, thus protecting the body against the development of numerous chronic diseases, such as cardiovascular and neurodegenerative diseases, diabetes and tumor diseases [3]. In addition, these fruits are characterized by an impressive amount of crude fiber, containing between 7.04 and 22.20 g of total dietary fiber per 100 g of fresh ripe strawberry tree fruit [4,5,6,7]. From a technological point of view, they could be highlighted as an interesting raw material for the production of functional food.

In response to solving global crises with a focus on sustainability in food production and processing, the fourth industrial revolution or Industry 4.0 was initiated by a combination of information and communication solutions that is now visible in numerous sectors where, thanks to digitalization, it is significantly changing the way new products are designed or manufactured [8]. The guidelines for Industry 4.0 particularly emphasize the use of non-thermal processing technologies together with additive technologies, with three-dimensional printing (3DP) leading the way in food technology [9]. Three-dimensional printing is a relatively fast process of additive, layer-by-layer production in which computer models enable the production of 3D products in various shapes. This technology is already being explored for functional food design, so its application to various raw materials such as broccoli and carrots [10]; a blend of calcium caseinate powder, starch and medium-chain triglyceride powder [11]; a betaine-enriched oat-based blend [12]; orange by-products [13,14]; a gluten-free cereal blend [15,16]; and a pumpkin blend [17] has been investigated. However, fruit matrices for the 3DP are particularly challenging as it is difficult to produce a functional product that meets the nutritional, biological, textural and sensory requirements [18].

In a previous work, the influence of different amounts (10%, 15% and 20%) and types of starch (wheat vs. corn) and the 3DP programs on the stability of bioactive compounds, antioxidant capacity and textural properties of the strawberry-based 3D-printed product was investigated [19]. A similar study was also conducted with 3D-printed *A. unedo* products to optimize the 3DP technology for this fruit material [2]. Both studies showed a significant influence of the amount and type of starch as well as the 3DP processing parameters on the stability of the bioactive compounds, antioxidant capacity and textural properties. However, due to its chemical and textural properties, *A. unedo* proved to be an excellent raw material that can serve as an excellent basis for the development of various formulations of 3D functional products—in contrast to strawberry, which poses a major challenge due to its high water content.

Based on all the above, the aim of this work was to investigate the possibility of combining two fruit bases, *A. unedo* and strawberry, in the production of 3D-printed functional snacks. The idea is to improve the nutritional, biological, rheological and sensory properties of innovative functional 3D snack products by combining these two fruits. To this end, the influence of different amounts of wheat starch (6%, 8% and 10%) and the type of 3D printing program on the content of bioactive compounds (total phenolic content, total hydroxycinnamic acids, total flavonols and condensed tannins), pigments (monomeric anthocyanins, total carotenoids, chlorophyll A and chlorophyll B), antioxidant capacity (DPPH and FRAP), color and rheological properties of the 3D-printed snacks was investigated. The 3D-printed snack sample characterized by its best bioactive potential and rheological properties was selected for the continuation of the sensory acceptability study, where the addition of eight different sweeteners in two different concentrations was tested.

## 2. Materials and Methods

### 2.1. Fruit Material

The samples for the 3D snacks were produced from the fruits of strawberries (*Fragaria ananassa* × Duch., cv. ‘Albion’) and strawberry trees (*Arbutus unedo* L.). The strawberries were supplied by the company Jagodar-HB d.o.o. (Donja Lomnica, Zagreb County, Croatia). The fruits of *A. unedo* were collected in the southern part of the island of Lošinj (Primorsko-Goranska County, Croatia). After delivery to the laboratory, the fruits were washed, cleaned, dried and stored in plastic bags at −18 °C until the experiments. Wheat starch (Denes Natura Kft., Pécs, Hungary) was used as a hydrocolloid carrier to prepare the fruit mixture for 3D printing. All the sweeteners used for the sensory evaluation were purchased from the local market.

### 2.2. Preparation of Fruit Material for 3D Printing

Each of the fruits was thawed, homogenized with a Cordys SB-1 blender (MS Industrial Ltd., Hong Kong, China) and then mixed in a 1:1 ratio (*w*/*w*). The wheat starch (6%, 8% and 10% *w*/*w*) was added to the prepared fruit mixture to achieve an appropriate viscous texture. The mixture was then heated to 65 °C with constant stirring on an LLG-uniSTIRRER 7 magnetic stirrer (Lab Logistics Group GmbH, Meckenheim, Germany) to obtain a mixture with suitable viscosity for 3D printing.

### 2.3. Three-Dimensional Printing of Fruit Snacks

**Figure 1:**
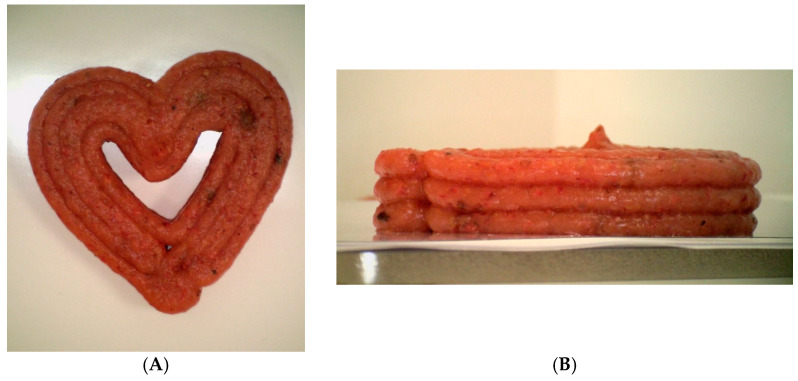
Designed heart shape for 3D printing: top view (**A**) and side view (**B**).

A Foodini 3D printer (Natural Machines, Barcelona, Spain) with a nozzle diameter of 4 mm was used for the 3DP of the functional snacks. A 3D heart shape (Figure 1A) with three layers (Figure 1B) was designed using the Foodini Creator computer program. The 3DP was performed with two different programs (P1 and P2), differing in the printing speed (8000 mm min^−1^ vs. 14,000 mm min^−1^), printing line thickness (3.5 mm vs. 3.4 mm), mixture flow rate (1.4 vs. 1.65 (dimensionless)) and nozzle height of the first layer (6 mm vs. 4.5 mm). The dimensions of the 3D-printed objects were 53 mm (length) × 51 mm (width) × 12 mm (height).

The design of the 3DP experiments is presented in Table 1.

### 2.4. Extraction of Antioxidant Bioactive Compounds

Ultrasound-Assisted Extraction (UAE) was performed to isolate the bioactive compounds from all the analyzed samples using a processor (UP400St Hielscher Ultrasound Technology, Teltow, Germany) equipped with a DN22 titanium sonotrode (546 mm^2^) [20]. In brief, 1% formic acid in 80% methanol (*v*/*v*) was used as the extraction solvent. A total of 10 g of the sample was placed in an Erlenmeyer flask and 40 mL of the extraction solvent was poured over it. The UAE was performed at 50% amplitude and 100% pulse for 5 min. The extract was then filtered into a 50 mL flask and made up with the extraction solvent.

The extracts obtained were used in the procedures for the spectrophotometric determination of the total phenols, total flavonoids, hydroxycinnamic acids, flavonols, monomeric anthocyanins and condensed tannins as well as for the analysis of the antioxidant activities using the DPPH and FRAP methods. All the measurements were carried out in duplicate.

### 2.5. Determination of Polyphenolic Compounds

All the 3D-printed snacks were spectrophotometrically analyzed for the polyphenol content, pigments and antioxidant capacity using a UV–vis spectrophotometer (LLG-uniSPEC 2 spectrophotometer, Buch and Holm, Meckenheim, Germany).

#### 2.5.1. Determination of Total Phenolic Content (TPC)

A total of 400 µL of the extract, 400 µL of the Folin Ciocalteu reagent and 4 mL of a 7.5% sodium carbonate solution were pipetted successively into the test tubes. The reaction mixture was allowed to stand at room temperature for 20 min. The absorbance was measured at 725 nm. The calibration curve was prepared with the solutions of different concentrations of gallic acid (10–250 mg L^−1^). The equation of the calibration curve used to determine the total phenolic content was:

y = 0.0078x − 0.0032,

where

y—the absorbance of the sample at 725 nm;

x—the concentration of gallic acid (mg L^−1^). The TPC results were expressed as mg gallic acid equivalents (GAEs) per 100 g sample [21].

#### 2.5.2. Determination of Total Flavonoids (TF)

Briefly, 0.5 mL of the extract, 1.5 mL of 96% ethanol, 0.1 mL of 10% aluminum chloride, 0.1 mL of 1 M potassium acetate and 2.8 mL of distilled water were added into a glass tube. A blank sample was prepared in the same way, but an extraction solvent was used instead of the extract and the same volume of distilled water (0.1 mL) was added instead of 10% aluminum chloride. The reaction mixture was then allowed to stand for 30 min and the absorbance was measured at 415 nm. Quercetin standard solutions (10–200 mg L^−1^) were used to generate a calibration curve and the results were expressed as mg quercetin equivalents (QEs) per 100 g of sample [22].

#### 2.5.3. Determination of Total Hydroxycinnamic Acids (HCAs) and Total Flavonols (FLs)

A total of 250 μL of the extract, 250 μL of 1 g L^−1^ HCl in 96% ethanol and 4.55 mL of 2 g L^−1^ HCl were pipetted into a glass tube. The absorbance was then measured at 320 nm for the HCA and at 360 nm for the FL. The HCA content was calculated from the calibration curve obtained from the solutions of different concentrations of chlorogenic acid (10–600 mg L^−1^), and the results were expressed as the mg chlorogenic acid equivalent (CAE) per 100 g sample. The FL content was calculated from the calibration curve obtained from the solutions of different concentrations of quercetin (10–600 mg L^−1^), and the results were expressed as the mg quercetin equivalent (QE) per 100 g of sample [23].

#### 2.5.4. Determination of Condensed Tannins (CTs)

In total, 2.5 mL of 1% vanillin, 2.5 mL of 25% H_2_SO_4_ solution and 1 mL of the extract were pipetted into a glass tube. The mixture was mixed and allowed to stand at room temperature for 10 min. Then, the absorbance was measured at 500 nm. The calibration curve was prepared from the catechin solutions of different concentrations (10–120 mg L^−1^) and the results were expressed as the mg catechin equivalent (CE) per 100 g of sample [24].

### 2.6. Determination of Pigments

#### 2.6.1. Determination of Total Monomeric Anthocyanins (ANTs)

The anthocyanin content was determined using the pH differential method [25]. In total, 1 mL of the extract was mixed with 4 mL of potassium chloride buffer pH 1.0 (0.025 M) and also 1 mL of the extract was mixed with 4 mL of sodium acetate buffer pH 4.5 (0.4 M). After 20 min, the absorbance of the reaction mixtures was measured at 520 nm and 700 nm. The ANT content was expressed as the mg cyanidin-3-glucoside equivalent (Cy-3-Glc) per 100 g of sample.

#### 2.6.2. Determination of Total Carotenoids (CARs), Chlorophyll A (CHL A) and Chlorophyll B (CHL B)

The determination of the CAR, CHL A and CHL B was performed using the previously established method [26]. In total, 5 g of the sample was placed in an Erlenmayer flask and 25 mL of the extraction solvent (80% acetone, *v*/*v*) was added. The prepared mixture was then extracted in an ultrasonic bath (DT 514 H SONOREX DIGITEC, 13.5 L, 860 W, 40 kHz, Bandelin electronic, Berlin, Germany) at 50 °C for 30 min. After the extraction, the samples were filtered into 25 mL volumetric flasks and filled up to the mark with 80% acetone. The absorbance was measured at 470 nm, 646.8 nm and 663.2 nm. The concentrations of the CAR, CHL A and CHL B were calculated according to the formula from the literature [26] and expressed in mg 100 g^−1^ of sample.

### 2.7. In Vitro Antioxidant Capacity (AOC)

#### 2.7.1. DPPH (2,2-Diphenyl-1-picrylhydrazyl) Scavenging Activity Assay

The antiradical activities of the bioactive antioxidants were determined using the DPPH method [27]. In brief, 1.5 mL of the extract and 3 mL of 0.5 mM DPPH solution were pipetted into a test tube and kept at room temperature in the dark for 20 min. The absorbance was then measured at 517 nm. A calibration curve was constructed from different concentrations of Trolox solutions (10–150 µM) and the results were expressed as the µmol Trolox equivalent (TE) per 100 g of sample.

#### 2.7.2. FRAP (Ferric-Reducing Antioxidant Power) Assay

The FRAP method was performed according to the literature protocol [28]. A total of 600 μL of extract and 4500 μL of FRAP reagent (prepared from acetate buffer (0.3 M), 2.5 mL of TPTZ reagent (2,4,6-tris-2-pyridyl-s-triazine; 10 mM) and 2.5 mL of iron (III) chloride (20 mM) in a 10:1:1 ratio) were pipetted into glass tubes, mixed and thermostatted at 37 °C for 10 min. The absorbance was then measured at 593 nm. A calibration curve was constructed from different concentrations of Trolox solutions (10–150 µM) and the results were expressed as the mmol Trolox equivalent (TE) per 100 g of sample.

### 2.8. Determination of Rheological Properties of 3DP Snacks

#### 2.8.1. Texture Analysis

An evaluation of the rheological properties was conducted using the TA.HD plus Texture Analyser (Stable Micro System, Godalming, UK), applying two tests: the forward extrusion test and penetration test.

##### Forward Extrusion Test

The testing was conducted using an extrusion set (cylindrical sample container and a piston disc). The base disk is set at the bottom of the sample container with a central opening of 3 mm in diameter. The parameters are as follows: test speed, 1 mms^−1^; outgoing speed, 10 mms^−1^; and extrusion distance, 20 mm with trigger force 10 g.

The testing measures the compression force required for the piston to extrude the 3D-printed sample through the opening of the disk. Each sample batch was tested three times, and all the tests were performed at room temperature. The results are expressed as the mean extrusion force (F) (firmness) and work (W) required for extruding the samples.

##### Penetration Test

A spherical probe with a diameter of 4 mm was utilized during the test. The parameters were set to the following operating speeds: test speed, 0.5 mms^−1^; outgoing speed, 10 mms^−1^; and deformation distance, 6 mm with trigger force 2 g. Three parallel measurements were conducted at room temperature, and the results are presented as the maximum force (F_p_) (hardness) and work (W_p_).

#### 2.8.2. Dimension Measurements

The dimensions of the samples are expressed in terms of the geometric parameters (length × width × height). The differences in the measured values were determined using a digital caliper with an accuracy of 0.01 mm. The minimal differences in the sample dimensions using different programs without starch content are program 1: 58 mm × 58 mm × 9.4 mm and program 2: 62 mm × 59 mm × 9 mm (length × width × height).

#### 2.8.3. Particle Size Distribution

The particle size distribution of the samples in the Hydro 2000S system was determined using the laser diffraction method (Malvern Masterseizer 2000, Malvern Instruments Ltd., Worcestershire, UK). Within the cylinder, 10 g of dissolved sample was dispersed in 30 mL of distilled water, ensuring the homogeneity of the solution for the measurement and achieving a minimal degree of obscuration. The particle diameters were expressed over D (3.2); D (4.3); d (0.1); d (0.5); and d (0.9).

### 2.9. Determination of Instrumental Color

Color measurements were carried out for each trial using a Konica Minolta Spectrophotometer (CM-700d, Konica Minolta, Tokyo, Japan), which featured a D65 10° standard observer light source and a target mask CM-A183 with an 8 mm aperture, and a glass-covered cone. In each trial, the 3D-printed sample was compressed under the target mask of the spectrophotometer and the colorimetric parameters (L*, a* and b*) were measured. The color change (∆E), chroma (C) and hue (H*) were calculated using the provided formulas:

(1) $$\Delta E_{\mathrm{ab}}^{*}=\sqrt{\Delta L^{*2}+\Delta a^{*2}+\Delta b^{*2}}$$

(2) $$C^{*}=\sqrt{a^{*2}+b^{*2}}$$

(3) $$H^{*}=\tan^{-1}\left(\frac{b^{*}}{a^{*}}\right)$$

where all ΔL*^2^, Δa*^2^ and Δb*^2^ were calculated on the differences between the control and 3D-printed samples. All the measurements were conducted in triplicate.

### 2.10. Sensory Evaluation of 3D-Printed Snacks

All the 3D-printed snacks were sensory-evaluated using the Quantitative Descriptive Analysis (QDA) method [29]. Based on the results obtained in the determination of the stability of the bioactive antioxidants, pigments and rheological properties by 3DP technology, a sample characterized by the best results was selected and a sensory evaluation was carried out with it. For this purpose, the addition of 8 different sweeteners was tested in 2 concentration levels (Table 2), which were determined in a preliminary sensory evaluation.

A team of 18 sensory panelists rated the sensory attributes using a line intensity scale, with the scores assigned on a scale of 0–7 to indicate the relative intensity of each attribute, with 0 indicating the complete absence of the sensory attribute and 7 indicating a very pronounced attribute. The samples were served in coded Petri dishes. A total of 12 sensory descriptors were evaluated, which included the following attributes: (i) color—intensity of orange color; (ii) odor—strawberry odor, off-odor; (iii) aroma—strawberry flavor, strawberry tree fruit flavor, off-flavor; (iv) taste—sweet taste, sour taste, harmony taste, off-taste; and (v) texture—homogeneity, glossy appearance.

### 2.11. Statistical Analysis

A multivariate analysis of variance (MANOVA) with Tukey’s HSD was performed to simultaneously test the relationships between the dependent and categorical variables. The significance for all the tests was *p* ≤ 0.05. All the results were analyzed using Statistica software (v. 14.1) [30]. The results of the rheological properties obtained were analyzed using Statistica 12 software. The statistical significance of the influence of the process parameters on the parameters of the descriptive statistics was determined by conducting a MANOVA. The results were considered statistically significant if *p* ≤ 0.05 (95% significance level).

## 3. Results and Discussion

### 3.1. Characterization of Polyphenolic Compounds, Pigments and Antioxidant Capacity in 3D-Printed Snacks

Table 3 shows the results for the influence of four different proportions of wheat starch, namely, 0, 6, 8 and 10%, on the content of the polyphenolic compounds in the 3D samples. Considering all the phenolic compounds determined, condensed tannins were the most abundant (150.56 ± 3.31 mg 100 g^−1^), followed by hydroxycinnamic acids (74.79 ± 2.03 mg 100 g^−1^), flavonols (49.84 ± 1.31 mg 100 g^−1^) and total flavonoids (9.86 ± 0.16 mg 100 g^−1^). These results are consistent with the results of previous studies on the bioactive composition of strawberries [31].

When considering the influence of starch content, it can be seen that the content of total phenolic compounds, hydroxycinnamic acids and flavonols decreases with increasing starch content (0–10%). These results are consistent with previous reports [2], in which an increase in starch content from 4 to 8% led to a decrease in the content of total phenolic compounds. The results obtained indicate a negative effect of increasing starch content on the content of bioactive compounds, which is rather expected due to the higher content of non-phenolic compounds, i.e., starch. However, no such correlation was observed for the total flavonoids and condensed tannins. In the case of condensed tannins, it was found that there was no difference in their concentration when different proportions of starch were added. An unusual trend was observed for the content of total flavonoids, where the highest concentrations were recorded in samples without added starch (0%) and in samples with 8% starch. However, when the starch content was further increased to 10%, the TF content had the lowest value. The reason for this could be interactions within the matrix of the mixture, as well as interference in the spectrophotometric assay for the total flavonoids caused by non-flavonoid compounds. Namely, starch can react with other components of the mixture, such as some bioactive compounds, which can lead to an increase in their availability. In addition, the presence of starch can affect the solubility of bioactive compounds, which also affects their increase in concentration [32].

The 3D printing of the samples was carried out with two different programs (P1 and P2). These programs differed in terms of the 3DP speed, the print line thickness, the flow rate of the mixture and the nozzle height of the first layer. The only significant influence of the 3DP program was observed for the HCA content as a lower amount of HCA was found in the samples printed with program 2 compared to program 1. These two programs may have a different influence on the bioactive compound content as the 3DP process parameters, such as the nozzle movement speed, pressure and flow rate, differ between these two programs [33]. Program 2 could lead to better preservation of the bioactive compounds, as it has a higher flow rate of the mixture and a higher printing speed compared to program 1. As a result, the processing time is shorter, which means that the bioactive compounds are exposed for less time to conditions that can degrade them. The influence of printing process parameters, i.e., printing programs, on the structural quality of 3D-printed products has already been investigated, but there is not yet enough data on their influence on the stability of bioactive compounds in a 3D-printed product.

The influence of the addition of starch and the 3D programs was also observed on the influence of the stability of the pigments and the antioxidant capacity in the 3D samples (Table 4). The highest concentrations of anthocyanins and chlorophylls were determined in the samples without added starch, while the starch content of 6% had the most favorable influence on the stability of the carotenoids. Furthermore, when the starch content was increased by 6–10%, no significant differences were found in the content of the anthocyanins, chlorophylls A and chlorophylls B in the 3DP samples. A different trend was observed for the carotenoids. Increasing the starch content had a negative effect on the stability of the carotenoids. Increasing the starch content from 0 to 10% had no significant effect on the DPPH values; while higher FRAP values were obtained for the 3D samples with 0–8% added starch, the samples with 10% had the lowest values.

Bebek Markovinović et al. [19] also observed a decrease in the content of carotenoids and antioxidant activity values determined by the FRAP method when the starch content in the 3DP strawberry-based products was increased to over 15%. The reason for the initial deviations from this trend in the carotenoid and FRAP values may be that this study used a strawberry and strawberry tree fruit-based blend, which has different rheological properties than a strawberry blend. Ultimately, the overall result of a decrease in the bioactive compound content was to be expected, as a printing mixture with higher starch content has a lower proportion of the fruit component, which is the source of the bioactive compounds. There is also the possibility of interactions between bioactive compounds and starch in 3DP products with higher starch content, which may result in lower levels of the bioactive compound. It is hypothesized that phenolic hydroxyl groups may interact with starch by forming non-covalent bonds, such as hydrogen bonds, and that electrostatic and ionic interactions may occur, leading to the formation of complex compounds. However, the addition of starch, which triggers such chemical reactions, can lead to an improvement in the nutritional and physico-chemical properties of the product and the digestibility of the starch [34].

The 3DP programs had no effect on the stability of the pigments and on the antioxidant capacity. The reason for this could be that the process parameters of these two programs are not sufficiently different to cause statistically significant changes in the abovementioned parameters. It can also be assumed that some other bioactive compounds, which were not considered in this study, contribute to the antioxidant capacity, and the differences in the 3DP process parameters by the two programs had no effect on them.

Table 5 shows the correlations between the investigated bioactive compounds and the antioxidant capacity (DPPH and FRAP). The TPC correlates positively with the content of all the bioactive compounds, most strongly with the HCA and least with the TF. They also correlate with the DPPH. The other bioactive compounds examined also show a positive correlation with similar chemical structures. When looking at the antioxidant capacity, the DPPH correlates significantly with the TPC, HCA, FL and CAR, while there are no significant correlations with the other bioactive compounds. On the other hand, the FRAP does not correlate significantly with any of the bioactive compounds or DPPH. This suggests that the FRAP values are related to some other bioactive compounds that were not studied. Andrés et al. [35] showed that the FRAP capacity in a smoothie of orange juice, papaya juice, melon juice, carrot puree and skim milk correlated significantly with ascorbic acid and polyphenolic compounds but not with carotenoids.

### 3.2. Characterization of Rheological Properties in 3D-Printed Snacks

#### 3.2.1. Texture Analysis (Forward Extrusion and Penetration Test)

The results presented in Table 6 show that by applying program 2 and increasing the starch content in the printing mixture from 6% to 8%, the extrusion force does not change significantly. However, the total flow resistance through the cylinder increases considerably during the 3DP of the mixture with a starch content of 10%. The substantially higher flow resistance observed during the extrusion of the samples with the maximum starch content results from increased sample firmness. This is consistent with the findings of the studies conducted by Feng et al. [36], Dong et al. [37] and Yang et al. [38,39], who found that the starch content in the printing mixture is proportional to the sample firmness. By applying program 1, which has a lower printing speed, the extrusion force decreases as the starch content in the mixture increases. A higher starch content results in a more consistent sample structure, increasing the flow index.

Consequently, the sample’s fluidity increases and the total resistance during extrusion through the cylinder container decreases. One possible explanation for these results, whose statistical significance is shown in Table 7, is the significant influence of the apparent viscosity value indicating the pseudoplastic character of the extruded samples. Samples 3D printed with program 1 were expected to exhibit increased resistance with increasing starch content, but the predicted effect did not materialize due to changes in the rheological parameters. Accordingly, no significant deviations from the values of the extrusion force change were observed in the extrusion work values as the printing speed and starch content in the samples changed. The aforementioned are in accordance with the findings of the research by Bebek Markovinović et al. [2], which demonstrated that the force and work required for the extrusion of the mixture significantly depend on the starch content—specifically, increasing the starch content led to increased values of work and extrusion force. The extrusion work of samples, like the extrusion force, can be considered a relevant factor in determining the textural properties of samples, with the note that, in this case, the path (the distance travelled by the piston through the cylinder) is the main factor defining the total work or the area under the curve and, consequently, the maximum extrusion force, defining its initial and final firmness.

According to the results obtained from the forward extrusion test, the penetration test method did not affect significant deviations in the characterization of the mechanical properties. Because the test is performed with smaller probe contact areas where the sensitivity of force (F_p_) (hardness) sensing is more pronounced and stresses are significantly lower, differences in the sample strength are even less pronounced, considering the starch content.

#### 3.2.2. Dimension Measurements of 3D-Printed Snacks

An increase in the starch content and the application of different printing programs did not result in statistically significant deviations in the dimensions of the printed samples. Table 8 shows the influence of the 3DP program on the dimensions of the 3DP fruit snack. The samples exhibit stability in all three observed geometry directions.

#### 3.2.3. Particle Size Distribution

An increase in the starch content has shown an influence on all the parameters of the particle size distribution. The translation of the medians toward the interval of smaller particle distribution is in correlation with a reduction in the diameter in the 90% of the total particle count (D 0.9). In accordance, the statistical significance can be attributed to the overall change in the values describing the surface area ratio of smaller diameter particles (D 3.2) compared to the volume ratio of larger diameter particles (D 4.3). From previous research by Bebek Markovinović et al. [2], it is evident that smaller diameter particles contribute to more excellent stability and durability of printed samples. Table 9 shows the influence of the printing process parameters on the particle diameters. The significance analysis confirmed that the starch content was the most important factor influencing the particle size distribution of the 3D-printed snacks.

**Figure 2:**
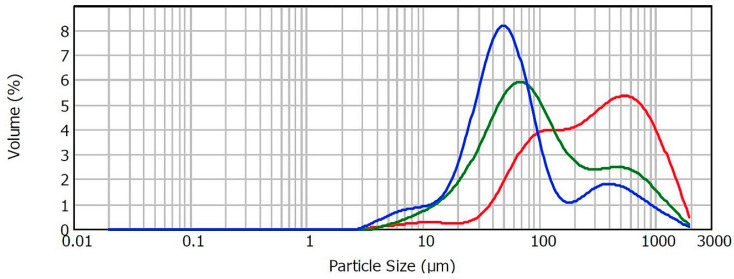
Volume size distribution of control sample (red), sample 4 with 6% (green) and sample 8 with 10% of starch (blue).

Comparing the control sample with the samples containing 6% and 10% starch, an increase in the volume fraction (relative frequencies) of the particles in the distribution interval of 17.38–120.22 μm is evident (Figure 2). The addition of starch contributed to the uniformity of the particle distribution curves compared to the control sample, which is characterized by a bimodal distribution. This indicates a greater stability of the samples with added starch in all ratios.

### 3.3. Characterization of Color Properties in 3D-Printed Snacks

Color plays an important role in the consumer’s perception of a particular product [40]. Table 10 shows the CIELab color parameters for the 3D-printed samples as a function of the starch content (0, 6, 8 and 10%) and printing program (P1 and P2).

The starch content significantly influenced the lightness value (L*) of the 3D-printed samples. The samples without added starch had the lowest L* value, i.e., they were the brightest. The highest L* value was recorded for the samples with a starch content of 6%, whereupon a further increase in the starch content to 8% led to a decrease. When the starch content was further increased to 10%, no significant difference was observed in the L* parameter. Considering that there was a significant difference in the L* values between the samples without and with the addition of starch, this darkening of the samples with the addition of starch could be due to the short-term exposure to an elevated temperature by heating the fruit mixture to achieve the appropriate consistency for 3DP. During this brief heating, it is possible that a slight non-enzymatic degradation of the mixture by Maillard reactions occurred due to the elevated temperature [41].

The change in starch content (0–10%) in the 3D-printed samples had no significant effect on the color parameter a*. Because the parameter a* refers to the red color, the results obtained indicate that the addition of starch has no significant effect on the expression of the red color, which is a highly desirable sensory characteristic. In contrast, a significant influence of the starch content on the color parameter b* was found. The samples without added starch had the lowest b* value, which was close to the value of the samples with 10% added starch, i.e., the samples without starch and with 10% added starch did not differ significantly. The highest b* value was found in the samples with a starch content of 6%. Increasing the starch content from 6% to 8% decreased the b* value significantly but not less than the samples without starch and with the addition of 10% starch. In our previous studies on 3D-printed strawberry products, the a* and b* parameters ranged from 22.54 to 27.52 and 7.50 to 12.98, respectively [19], while they ranged from 19.76 to 32.78 and 18.24 to 27.90 for 3D-printed strawberry products [2]. As expected, our results of the mean a* and b* values of strawberry and strawberry tree fruit 3D-printed snacks were approximately at the mean values of the a* and b* color parameters of the strawberry and strawberry tree fruit 3D-printed snacks examined separately in the two previous studies (23.40 ± 0.25 and 15.49 ± 0.29, respectively).

The C* and H* values follow almost the same trend with the change in starch content. The addition of 6% starch led to a statistically significant increase in the C* and H* values, while a further addition of 8% starch caused a significant decrease. A further 10% increase in the starch content resulted in a significant decrease in the C* value, while there was no effect on the H* value. The C* and H values of 3DP strawberry snacks from previous studies ranged from 23.85 to 29.93 and 18.33 to 25.96, respectively [19], while the values of 3DP strawberry tree fruit snacks ranged from 27.09 to 41.89 and 35.82 to 44.63, respectively [2]. Like the a* and b* values, the mean C* and H* values of the 3DP strawberry and strawberry tree fruit snacks (28.07 ± 0.35 and 33.46 ± 0.31, respectively) were similar to the mean values of the C* and H* color parameters of the 3DP strawberry and strawberry tree fruit snacks examined separately in the two previous studies.

The color change (ΔE) in the 3D-printed products ranged from 0.00 ± 0.41 to 6.17 ± 0.41 depending on the added starch content. The 3D product with a starch content of 6% showed the highest color change, while further increasing the starch content by 8% caused a significant decrease in the ΔE. No significant difference in the ΔE value was observed when the starch content was further increased by 10%. When we compare the L* and ΔE values, we find that they follow the same trend. In both cases, the darkest samples were those with the addition of 6% starch. It is possible that here the color of the fruit pulp was degraded by the short-term heating due to Maillard reactions [41]. Except for the samples with 6% added starch, all the others had a ΔE value of less than 6, which defines these differences as appreciable differences. Only the addition of 6% starch causes ΔE values above 6, which defines them as large differences [42]. The average ΔE value was 3.26 ± 0.60, and in general, the color differences in the 3D-printed strawberry and strawberry tree fruit products were acceptable.

The 3DP programs had no statistically significant effect on any color parameter (L*, a*, b*, C*, H and ΔE). Because the choice of program had no statistically significant influence on the content of the bioactive compounds (with the exception of HCA), including the content of anthocyanins, it is to be expected that no significant changes in the color parameters were observed under the influence of the different program parameters.

### 3.4. Characterization of the Sensory Properties of 3D-Printed Snacks

Sample 3, which contained 6% starch and was prepared with program 1, was selected for the sensory evaluation as it proved to be the best in terms of the stability of the bioactive compounds and antioxidant capacity. The 3D-printed snacks prepared with eight different sweeteners in two different concentrations (16 samples) and the control samples (without sweeteners) were evaluated using twelve sensory descriptors (Table 11). In the first part of the table, all the samples are compared with regard to the type and amount of added sweetener, while in the second part of the table, the samples are grouped according to the type of added sweetener.

The type of sweetener added and the level of concentration (lower vs. higher concentration) had no significant influence on the sensory properties with the exception of sweetness and harmonious taste. Therefore, no specific correlation was found between the color, taste and odor characteristics compared to the control samples. Significant differences were only found with regard to sweetness and harmonious taste. The perception of harmonious taste implies the relationship between sweetness and acidity [43] and represents a sensory descriptor that encompasses the general preference for the food product [44]. The samples C2, D2, E2, F2, H2 and I2 with a higher content of sweeteners showed a higher sweetness than their counterparts with a lower addition of sweeteners C1, D1, E1, F1, H1 and I1. As expected, the control sample had the lowest intensity of sweetness and differed significantly from the sweetened samples in almost all the comparisons. In addition, the greatest differences in harmonious taste were observed between samples D1 and D2 with the addition of birch sugar, E1 and E2 with the addition of erythritol and H1 and H2 with the addition of agave syrup. The aforementioned samples with a higher sweetener content had a higher harmonicity than their parallel samples with a lower sweetener content.

In the second part of Table 11, the samples are observed according to the type of added sweetener, without observation at the two concentration levels in which they were added. Statistically significant differences between the samples were recorded in the sensory descriptors, such as strawberry odor, sweet taste, harmony taste and glossy appearance.

Sample E with the addition of erythritol had the least strawberry odor, while samples B, F and H had the strongest intensity of strawberry odor. The addition of saccharose, maple syrup and agave syrup in the mentioned samples had a certain positive effect on the strawberry odor.

The control sample without added sweetener had the lowest sweetness, while the samples with added saccharose (B), fructose (C), maple syrup (F), agave syrup (H) and stevia and erythritol (I) had the highest sweetness. It is not surprising that after the sample with added saccharose (B), the sample with added stevia and erythritol (I) had the highest sweetness. The sweetness of stevia comes from the steviol glycosides stevioside and rebaudioside A, which are 250 to 400 times sweeter than saccharose and do not respond to temperature and pH changes during processing [45]. The main disadvantage of using stevia in the production of functional foods is undesirable sensory properties such as a bitter and/or metallic taste [46], which was not the case here, most likely because stevia was chosen in combination with erythritol. Furthermore, the addition of sweeteners led not only to an increase in sweetness but also to an increase in the harmony of the taste, so that samples B, C and H showed the most pronounced harmonious characteristics.

The samples with the addition of saccharose and fructose, i.e., samples B and C, had the most expressive characteristic of a glossy appearance, while the other samples, with the exception of sample E, were most similar to control sample A. Ultimately, the results obtained show that functional 3D-printed snack products can be sensory-enhanced by the addition of appropriate sweeteners, which opens a perspective for exploring new formulations of 3D-printed fruit-based functional foods.

## 4. Conclusions

This study investigated the effects of starch content and 3D printing programs on the stability of polyphenolic compounds, pigments, antioxidant activity, color and rheological properties as well as sensory characteristics of 3D-printed snacks based on strawberries and strawberry tree fruits. The results showed a decrease in the total phenolic compounds (19.86%), hydroxycinnamic acids (23.65%) and flavonols (23.91%) with increasing starch content, while total flavonoids and condensed tannins showed different trends. The 3D printing programs showed no significant influence on most bioactive compounds, with the exception of hydroxycinnamic acids, whose content was 4.35% higher in the samples printed with program 1. This study also highlighted the influence of starch content on the pigments and antioxidant capacity, with different effects observed depending on the starch content. The color parameters were significantly affected by variations in the starch content, while the 3D printing programs had no significant effect.

This study investigated the rheological properties of 3D-printed snacks, focusing on texture, dimensions and particle size distribution. A higher starch content led to a higher extrusion force and flow resistance, enhancing sample firmness. However, the penetration tests showed only minimal effects on the mechanical properties. The dimensional measurements remained stable at different starch contents and printing programs. The particle size distribution shifted toward smaller particles with higher starch content, which improved the rheological stability of the sample.

Although process parameters such as the addition of starch and the variation in 3D programs significantly affect the CIEL*a*b* color parameters, the color change was generally satisfactory with no noticeable and/or appreciable difference. The sensory evaluation of 3D-printed snacks with different sweeteners provided interesting results. The differences in the sweetener content had no significant effect on the color, taste or odor descriptors, with the exception of sweetness and harmony. The addition of sweeteners not only increased the sweetness but also had a positive effect on the harmony of the flavor, which was particularly evident in the samples with sucrose, fructose and agave syrup. These results underline the potential of different sweeteners to improve the sensory properties of functional 3D-printed snacks.

In summary, the results obtained indicate the great potential of using fruit bases in the production of functional 3D-printed snacks, which, thanks to their good nutritional and biological potential and their rheological properties when using natural sweeteners, represent an excellent basis for the further development of functional personalized nutrition.

## Figures and Tables

**Table 1:** Experimental plan for 3DP of fruit snacks.

Sample ID	Starch Content (%)	3D Program
1	0	P1
2	0	P2
3	6	P1
4	6	P2
5	8	P1
6	8	P2
7	10	P1
8	10	P2

**Table 2:** Experimental design for the sensory evaluation of 3D-printed snacks.

Simpe ID	Sweeteners	Sweetener Content (%)
A	Control sample	Without sweetener
B1	Saccharose	6.1
B2	Saccharose	9.1
C1	Fructose	7.1
C2	Fructose	8.9
D1	Birch sugar (xylitol)	5.6
D2	Birch sugar (xylitol)	8.5
E1	Erythritol	3.2
E2	Erythritol	4.7
F1	Maple syrup	5.5
F2	Maple syrup	8.7
G1	Date syrup	5.2
G2	Date syrup	7.1
H1	Agave syrup	6.7
H2	Agave syrup	10.2
I1	Stevia and erythritol	2.5
I2	Stevia and erythritol	3.9

**Table 3:** Relationship between 3DP parameters and the content of bioactive compounds in the 3D-printed snacks.

Variable	n	TPC	HCAs	FLs	TFs	CTs
Starch level		p≤0.01 ^†^	p≤0.01 ^†^	p≤0.01 ^†^	p≤0.01 ^†^	p≤0.01 ^†^
0%	4	429.50 ± 5.87 ^a^	84.99 ± 0.73 ^a^	55.41 ± 0.54 ^a^	10.66 ± 0.22 ^a^	171.33 ± 1.66 ^a^
6%	4	392.96 ± 5.87 ^b^	78.16 ± 0.73 ^b^	52.55 ± 0.54 ^b^	9.65 ± 0.22 ^b^	141.87 ± 1.66 ^b^
8%	4	353.60 ± 5.87 ^c^	71.11 ± 0.73 ^c^	49.25 ± 0.54 ^c^	9.90 ± 0.22 ^a,b^	141.41 ± 1.66 ^b^
10%	4	344.21 ± 5.87 ^c^	64.89 ± 0.73 ^d^	42.16 ± 0.54 ^d^	9.22 ± 0.22 ^b^	147.59 ± 1.66 ^b^
3DP Program		p=0.43 ^‡^	p≤0.01 ^†^	p=0.05 ^‡^	p=0.17 ^‡^	p=0.34 ^‡^
Program 1	8	382.48 ± 4.15 ^a^	76.45 ± 0.52 ^a^	50.47 ± 0.38 ^a^	9.69 ± 0.15 ^a^	149.71 ± 1.17 ^a^
Program 2	8	377.65 ± 4.15 ^a^	73.12 ± 0.52 ^b^	49.22 ± 0.38 ^a^	10.02 ± 0.15 ^a^	151.39 ± 1.17 ^a^
Dataset average	16	380.07 ± 9.09	74.79 ± 2.03	49.84 ± 1.31	9.86 ± 0.16	150.56 ± 3.31

Results are expressed as mean ± standard error. Values represented with different letters are statistically different at *p* ≤ 0.05; ^†^ significant factor in multifactor analysis; and ^‡^ not significant factor in multifactor analysis. TPC—total phenolic content (mg GAE 100 g^−1^); HCAs—hydroxycinnamic acids (mg CAE 100 g^−1^); FLs—flavonols (mg QE 100 g^−1^); TFs—total flavonoids (mg QE 100 g^−1^); and CTs—condensed tannins (mg CE 100 g^−1^).

**Table 4:** Relationship between 3DP parameters and the content of pigments and antioxidant capacity in the 3D-printed snacks.

Variable	n	ANT	CAR	CHLA	CHLB	DPPH	FRAP
Starch level		p≤0.01 ^†^	p≤0.01 ^†^	p≤0.01 ^†^	p≤0.01 ^†^	p=0.07 ^‡^	p=0.03 ^†^
0%	4	9.65 ± 0.16 ^a^	0.58 ± 0.002 ^b^	0.24 ± 0.01 ^a^	0.40 ± 0.01 ^a^	2.90 ± 0.07 ^a^	290.38 ± 0.35 ^a,b^
6%	4	7.75 ± 0.16 ^b^	0.62 ± 0.002 ^a^	0.11 ± 0.01 ^b^	0.18 ± 0.01 ^b^	3.00 ± 0.07 ^a^	289.95 ± 0.35 ^a,b^
8%	4	7.73 ± 0.16 ^b^	0.52 ± 0.002 ^c^	0.11 ± 0.01 ^b^	0.17 ± 0.01 ^b^	2.74 ± 0.07 ^a^	290.82 ± 0.35 ^a^
10%	4	8.09 ± 0.16 ^b^	0.48 ± 0.002 ^d^	0.12 ± 0.01 ^b^	0.19 ± 0.01 ^b^	2.73 ± 0.07 ^a^	288.94 ± 0.35 ^b^
3DP Program		p=0.64 ^‡^	p=0.11 ^‡^	p=0.12 ^‡^	p=0.19 ^‡^	p=0.15 ^‡^	p=0.73 ^‡^
Program 1	8	8.35 ± 0.11 ^a^	0.55 ± 0.001 ^a^	0.15 ± 0.004 ^a^	0.25 ± 0.01 ^a^	2.90 ± 0.05 ^a^	289.96 ± 0.25 ^a^
Program 2	8	8.27 ± 0.11 ^a^	0.55 ± 0.001 ^a^	0.14 ± 0.004 ^a^	0.23 ± 0.01 ^a^	2.78 ± 0.05 ^a^	290.08 ± 0.25 ^a^
Dataset average	16	8.31 ± 0.22	0.55 ± 0.01	0.14 ± 0.01	0.24 ± 0.03	2.84 ± 0.05	290.02 ± 0.26

Results are expressed as mean ± standard error. Values represented with different letters are statistically different at *p* ≤ 0.05; ^†^ significant factor in multifactor analysis; and ^‡^ not significant factor in multifactor analysis. ANT—monomeric anthocyanin (mg Cy-3-Glc 100 g^−1^); CAR—total carotenoid (mg 100 g^−1^); CHL A—total chlorophyll A (mg 100 g^−1^); CHL B—total chlorophyll B (mg 100 g^−1^); DPPH assay (µmol TE 100 g^−1^); and FRAP assay (mmol TE 100 g^−1^).

**Table 5:** Mutual correlations of bioactive compounds and antioxidant capacity.

	TPC	HCA	FL	TF	ANT	CT	CAR	CHL A	CHL B	DPPH	FRAP
TPC		0.92 *	0.84 *	0.56 *	0.71 *	0.69 *	0.75 *	0.77 *	0.77 *	0.51 *	0.32
HCA	0.92 *		0.91 *	0.66 *	0.63 *	0.61 *	0.78 *	0.69 *	0.69 *	0.50 *	0.41
FL	0.84 *	0.91 *		0.63 *	0.44	0.42	0.86 *	0.54 *	0.55 *	0.55 *	0.44
TF	0.56 *	0.66 *	0.63 *		0.63 *	0.64 *	0.43	0.64 *	0.65 *	0.13	0.28
ANT	0.71 *	0.63 *	0.44	0.63 *		0.90 *	0.22	0.93 *	0.92 *	0.25	0.09
CT	0.69 *	0.61 *	0.42	0.64 *	0.90 *		0.19	0.81 *	0.80 *	0.07	0.05
CAR	0.75 *	0.78 *	0.86 *	0.43	0.22	0.19		0.33	0.35	0.68 *	0.25
CHL A	0.77 *	0.69 *	0.54 *	0.64 *	0.93 *	0.81 *	0.33		1.00 *	0.26	0.20
CHL B	0.77 *	0.69 *	0.55 *	0.65 *	0.92 *	0.80 *	0.35	1.00 *		0.27	0.20
DPPH	0.51 *	0.50 *	0.55 *	0.13	0.25	0.07	0.68 *	0.26	0.27		−0.03
FRAP	0.32	0.41	0.44	0.28	0.09	0.05	0.25	0.20	0.20	−0.03	

* Correlations are significant at *p* < 0.05. TPC—total phenolic content (mg GAE 100 g^−1^); HCA—hydroxycinnamic acid (mg CAE 100 g^−1^); FL—flavonol (mg QE 100 g^−1^); TF—total flavonoid (mg QE 100 g^−1^); ANT—monomeric anthocyanin (mg Cy-3-Glc 100 g^−1^); CT—condensed tannin (mg CE 100 g^−1^); CAR—total carotenoid (mg 100 g^−1^); CHL A—total chlorophyll a (mg 100 g^−1^); CHL B—total chlorophyll b (mg 100 g^−1^); DPPH assay (µmol TE 100 g^−1^); and FRAP assay (mmol TE 100 g^−1^).

**Table 6:** Influence of 3DP processing parameters on textural properties of 3D-printed snacks.

Sample	3DP Program	Starch Content (%)	F (N)	W (Nmm)	F_p_ (N)	W_p_ (Nmm)
1	1	0	16.89	168.76	0.01	0.01
2	2	0	7.58	75.79	0.01	0.01
3	1	6	454.73	4545.01	0.07	0.06
4	2	6	54.08	540.43	0.05	0.05
5	1	8	112.82	1127.64	0.05	0.06
6	2	8	66.77	667.37	0.05	0.06
7	1	10	75.69	756.59	0.07	0.07
8	2	10	1003.08	10,025.82	0.06	0.04

F—mean extrusion force (firmness) (N); W—work (Nmm); F_p_—maximum force (hardness); and W_p_—work (Nmm).

**Table 7:** Influence of 3DP process parameters on texture, particle diameters and dimension in 3D-printed snacks expressed by *p*-value *.

Parameter	3DP Program	Starch Content (%)
F	0.725588	0.695556
W	0.725577	0.695550
F_p_	0.178047	0.158750
W_p_	0.236516	0.876531
D [3.2]	0.949818	0.003742 *
D [4.3]	0.660607	0.024213 *
d [0.1]	0.730548	0.029678 *
d [0.5]	0.616439	0.004638 *
d [0.9]	0.603835	0.037802 *
Length	0.147469	0.754530
Width	0.659869	0.795063
Height	0.122649	0.334880

* Results are statistically significant at *p* ≤ 0.05.

**Table 8:** Influence of 3DP processing parameters on the dimension of 3D-printed snacks.

Sample	3DP Program	Starch Content (%)	Length (mm)	Width (mm)	Height (mm)
1	1	0	58.88 ± 0.11	58.69 ± 0.22	9.40± 0.09
2	2	0	62.01± 0.17	59.94 ± 0.19	9.39± 0.12
3	1	6	54.85 ± 0.21	53.33 ± 0.53	12.55 ± 0.14
4	2	6	55.70 ± 0.15	53.57 ± 0.31	12.12 ± 0.15
5	1	8	55.06 ± 0.07	51.57 ± 0.23	13.47 ± 0.52
6	2	8	55.60 ± 0.32	53.99 ± 0.44	12.83 ± 0.32
7	1	10	53.73 ± 0.56	54.18 ± 0.09	13.50 ± 0.17
8	2	10	55.98 ± 0.37	53.09 ± 0.21	11.98 ± 0.26

The results are presented as an average value of triplicate measurements ± standard deviation.

**Table 9:** Influence of 3DP processing parameters on particle diameters in 3D-printed snacks.

Sample	3DP Program	Starch Content (%)	D [3.2] (μm)	D [4.3] (μm)	d [0.1] (μm)	d [0.5] (μm)	d [0.9] (μm)
1	1	0	120.24	429.11	62.18	297.58	1012.46
2	2	0	117.87	418.75	61.57	290.21	985.83
3	1	6	54.63	234.50	24.05	92.45	682.84
4	2	6	54.02	216.56	24.23	88.52	622.87
5	1	8	40.07	167.06	20.05	59.94	501.01
6	2	8	39.41	174.85	19.28	59.94	530.68
7	1	10	35.30	145.02	17.82	53.20	437.02
8	2	10	36.72	143.65	19.13	54.41	419.94

D [3.2]—surface weighted mean diameter (Sauter mean diameter); D [4.3]—volume weighted mean diameter (De Brouckere mean diameter); d [0.1]—10% of the volume distribution is below the observed diameter; d [0.5]—median diameter, 50% of the volume distribution is below and 50% is above the observed diameter; and d [0.9]—90% of the volume distribution is below the observed diameter.

**Table 10:** Relationship between 3DP parameters and the color parameters in the 3D-printed snacks.

Variable	n	L*	a*	b*	C*	H*	ΔE
Starch level		p≤0.01 ^†^	p=0.05 ^‡^	p≤0.01 ^†^	p≤0.01 ^†^	p≤0.01 ^†^	p≤0.01 ^†^
0%	4	41.43 ± 0.19 ^c^	22.92 ± 0.39 ^a^	14.44 ± 0.30 ^b^	27.09 ± 0.46 ^b^	32.23 ± 0.37 ^b^	0.00 ± 0.41 ^c^
6%	4	46.74 ± 0.19 ^a^	24.48 ± 0.39 ^a^	16.98 ± 0.30 ^a^	29.80 ± 0.46 ^a^	34.74 ± 0.37 ^a^	6.17 ± 0.41 ^a^
8%	4	44.79 ± 0.19 ^b^	23.45 ± 0.39 ^a^	15.70 ± 0.30 ^a,b^	28.22 ± 0.46 ^a,b^	33.80 ± 0.37 ^a,b^	3.89 ± 0.41 ^b^
10%	4	44.19 ± 0.19 ^b^	22.77 ± 0.39 ^a^	14.82 ± 0.30 ^b^	27.17 ± 0.46 ^b^	33.06 ± 0.37 ^a,b^	3.00 ± 0.41 ^b^
3DP Program		p=0.29 ^‡^	p=0.71 ^‡^	p=0.35 ^‡^	p=0.93 ^‡^	p=0.11 ^‡^	p=0.15 ^‡^
Program 1	8	44.18 ± 0.13 ^a^	23.48 ± 0.27 ^a^	15.34 ± 0.21 ^a^	28.05 ± 0.32 ^a^	33.13 ± 0.26 ^a^	3.59 ± 0.29 ^a^
Program 2	8	44.39 ± 0.13 ^a^	23.33 ± 0.27 ^a^	15.64 ± 0.21 ^a^	28.09 ± 0.32 ^a^	33.79 ± 0.26 ^a^	2.94 ± 0.29 ^a^
Dataset average	16	44.29 ± 0.50	23.40 ± 0.25	15.49 ± 0.29	28.07 ± 0.35	33.46 ± 0.31	3.26 ± 0.60

Results are expressed as mean ± standard error. Values represented with different letters are statistically different at *p* ≤ 0.05; ^†^ significant factor in multifactor analysis; and ^‡^ not significant factor in multifactor analysis. L*—lightness; a*—redness; b*—yellowness; C*—chroma; H*—hue; and ΔE—color change.

**Table 11:** Sensory comparison results of 3D-printed snacks with the addition of different sweeteners in two different concentrations.

Variable	n	Intensity of Orange Color	Strawberry Odor	Off-Odor	Strawberry Flavor	Strawberry Tree Fruit Flavor	Off-Flavor	Sweet Taste	Sour Taste	Harmony Taste	Off-Taste	Homogeneity	Glossy Appearance
Sample		p=0.81 ^‡^	p=0.76 ^‡^	p=0.99 ^‡^	p=0.07 ^‡^	p=0.99 ^‡^	p=0.41 ^‡^	p≤0.01 ^†^	p=0.14 ^‡^	p≤0.01 ^†^	p=0.67 ^‡^	p=0.71 ^‡^	p=0.09 ^‡^
A	13	5.85 ± 0.32 ^a^	5.38 ± 0.37 ^a^	1.23 ± 0.19 ^a^	4.46 ± 0.38 ^a^	4.15 ± 0.45 ^a^	1.15 ± 0.18 ^a^	2.46 ± 0.36 ^d^	4.69 ± 0.38 ^a^	3.69 ± 0.34 ^a,b^	1.15 ± 0.18 ^a^	5.15 ± 0.41 ^a^	5.77 ± 0.35 ^a^
B1	13	5.92 ± 0.32 ^a^	5.08 ± 0.37 ^a^	1.08 ± 0.19 ^a^	4.92 ± 0.38 ^a^	3.54 ± 0.45 ^a^	1.08 ± 0.18 ^a^	4.46 ± 0.36 ^a,b^	3.23 ± 0.38 ^a^	4.85 ± 0.34 ^a,b^	1.08 ± 0.18 ^a^	4.77 ± 0.41 ^a^	5.77 ± 0.35 ^a^
B2	13	6.08 ± 0.32 ^a^	5.00 ± 0.37 ^a^	1.23 ± 0.19 ^a^	4.85 ± 0.38 ^a^	3.46 ± 0.45 ^a^	1.08 ± 0.18 ^a^	5.00 ± 0.36 ^a,b^	2.92 ± 0.38 ^a^	4.85 ± 0.34 ^a,b^	1.08 ± 0.18 ^a^	5.15 ± 0.41 ^a^	6.08 ± 0.35 ^a^
C1	13	6.15 ± 0.32 ^a^	4.85 ± 0.37 ^a^	1.31 ± 0.19 ^a^	4.31 ± 0.38 ^a^	3.46 ± 0.45 ^a^	1.23 ± 0.18 ^a^	3.92 ± 0.36 ^a,b,c,d^	3.31 ± 0.38 ^a^	4.54 ± 0.34 ^a,b^	1.23 ± 0.18 ^a^	5.38 ± 0.41 ^a^	5.92 ± 0.35 ^a^
C2	13	5.92 ± 0.32 ^a^	4.77 ± 0.37 ^a^	1.23 ± 0.19 ^a^	4.31 ± 0.38 ^a^	3.23 ± 0.45 ^a^	1.23 ± 0.18 ^a^	4.77 ± 0.36 ^a,b^	2.92 ± 0.38 ^a^	4.85 ± 0.34 ^a,b^	1.08 ± 0.18 ^a^	5.31 ± 0.41 ^a^	6.15 ± 0.35 ^a^
D1	13	6.08 ± 0.32 ^a^	4.69 ± 0.37 ^a^	1.38 ± 0.19 ^a^	3.92 ± 0.38 ^a^	3.46 ± 0.45 ^a^	1.38 ± 0.18 ^a^	3.38 ± 0.36 ^b,c,d^	3.92 ± 0.38 ^a^	3.54 ± 0.34 ^b^	1.38 ± 0.18 ^a^	5.54 ± 0.41 ^a^	5.38 ± 0.35 ^a^
D2	13	6.23 ± 0.32 ^a^	4.92 ± 0.37 ^a^	1.08 ± 0.19 ^a^	4.15 ± 0.38 ^a^	3.46 ± 0.45 ^a^	1.08 ± 0.18 ^a^	4.00 ± 0.36 ^a,b,c,d^	3.54 ± 0.38 ^a^	4.31 ± 0.34 ^a,b^	1.23 ± 0.18 ^a^	5.54 ± 0.41 ^a^	5.46 ± 0.35 ^a^
E1	13	6.08 ± 0.32 ^a^	4.23 ± 0.37 ^a^	1.31 ± 0.19 ^a^	3.54 ± 0.38 ^a^	3.69 ± 0.45 ^a^	1.23 ± 0.18 ^a^	2.69 ± 0.36 ^c,d^	4.15 ± 0.38 ^a^	3.23 ± 0.34 ^b^	1.46 ± 0.18 ^a^	5.69 ± 0.41 ^a^	4.69 ± 0.35 ^a^
E2	13	6.15 ± 0.32 ^a^	4.15 ± 0.37 ^a^	1.23 ± 0.19 ^a^	3.46 ± 0.38 ^a^	3.54 ± 0.45 ^a^	1.31 ± 0.18 ^a^	3.54 ± 0.36 ^a,b,c,d^	4.08 ± 0.38 ^a^	3.85 ± 0.34 ^a,b^	1.23 ± 0.18 ^a^	5.46 ± 0.41 ^a^	4.62 ± 0.35 ^a^
F1	13	5.54 ± 0.32 ^a^	5.15 ± 0.37 ^a^	1.31 ± 0.19 ^a^	4.62 ± 0.38 ^a^	3.38 ± 0.45 ^a^	1.15 ± 0.18 ^a^	4.15 ± 0.36 ^a,b,c,d^	3.62 ± 0.38 ^a^	4.38 ± 0.34 ^a,b^	1.08 ± 0.18 ^a^	5.00 ± 0.41 ^a^	5.77 ± 0.35 ^a^
F2	13	5.38 ± 0.32 ^a^	4.92 ± 0.37 ^a^	1.15 ± 0.19 ^a^	5.15 ± 0.38 ^a^	3.38 ± 0.45 ^a^	1.15 ± 0.18 ^a^	4.62 ± 0.36 ^a,b^	3.31 ± 0.38 ^a^	4.69 ± 0.34 ^a,b^	1.23 ± 0.18 ^a^	5.38 ± 0.41 ^a^	5.69 ± 0.35 ^a^
G1	13	5.62 ± 0.32 ^a^	4.69 ± 0.37 ^a^	1.31 ± 0.19 ^a^	4.00 ± 0.38 ^a^	3.62 ± 0.45 ^a^	1.54 ± 0.18 ^a^	3.62 ± 0.36 ^a,b,c,d^	3.92 ± 0.38 ^a^	3.92 ± 0.34 ^a,b^	1.62 ± 0.18 ^a^	5.54 ± 0.41 ^a^	5.31 ± 0.35 ^a^
G2	13	5.62 ± 0.32 ^a^	5.00 ± 0.37 ^a^	1.38 ± 0.19 ^a^	4.46 ± 0.38 ^a^	3.38 ± 0.45 ^a^	1.38 ± 0.18 ^a^	4.00 ± 0.36 ^a,b,c,d^	3.54 ± 0.38 ^a^	4.31 ± 0.34 ^a,b^	1.38 ± 0.18 ^a^	5.69 ± 0.41 ^a^	5.54 ± 0.35 ^a^
H1	13	5.54 ± 0.32 ^a^	4.85 ± 0.37 ^a^	1.31 ± 0.19 ^a^	4.62 ± 0.38 ^a^	3.62 ± 0.45 ^a^	1.15 ± 0.18 ^a^	4.38 ± 0.36 ^a,b,c^	3.62 ± 0.38 ^a^	4.38 ± 0.34 ^a,b^	1.38 ± 0.18 ^a^	6.23 ± 0.41 ^a^	5.46 ± 0.35 ^a^
H2	13	5.54 ± 0.32 ^a^	5.08 ± 0.37 ^a^	1.23 ± 0.19 ^a^	5.08 ± 0.38 ^a^	3.69 ± 0.45 ^a^	1.15 ± 0.18 ^a^	5.15 ± 0.36 ^a^	3.38 ± 0.38 ^a^	5.23 ± 0.34 ^a^	1.23 ± 0.18 ^a^	5.85 ± 0.41 ^a^	5.54 ± 0.35 ^a^
I1	13	5.69 ± 0.32 ^a^	5.15 ± 0.37 ^a^	1.31 ± 0.19 ^a^	4.38 ± 0.38 ^a^	3.54 ± 0.45 ^a^	1.62 ± 0.18 ^a^	4.00 ± 0.36 ^a,b,c,d^	3.85 ± 0.38 ^a^	4.15 ± 0.34 ^a,b^	1.46 ± 0.18 ^a^	5.62 ± 0.41 ^a^	5.08 ± 0.35 ^a^
I2	13	5.77 ± 0.32 ^a^	5.15 ± 0.37 ^a^	1.15 ± 0.19 ^a^	4.69 ± 0.38 ^a^	3.54 ± 0.45 ^a^	1.62 ± 0.18 ^a^	4.53 ± 0.36 ^a,b^	3.85 ± 0.38 ^a^	4.38 ± 0.34 ^a,b^	1.38 ± 0.18 ^a^	6.00 ± 0.41 ^a^	5.08 ± 0.35 ^a^
Sample grouped		p=0.23 ^‡^	p=0.20 ^‡^	p=0.99 ^‡^	p≤0.01 ^†^	p=0.94 ^‡^	p=0.06 ^‡^	p≤0.01 ^†^	p=0.01 ^†^	p≤0.01 ^†^	p=0.27 ^‡^	p=0.23 ^‡^	p≤0.01 ^†^
A	13	5.85 ± 0.32 ^a^	5.38 ± 0.37 ^a^	1.23 ± 0.19 ^a^	4.46 ± 0.38 ^a,b^	4.15 ± 0.45 ^a^	1.15 ± 0.18 ^a^	2.46 ± 0.36 ^b^	4.69 ± 0.38 ^a^	3.69 ± 0.34 ^a,b^	1.15 ± 0.18 ^a^	5.15 ± 0.41 ^a^	5.77 ± 0.35 ^a,b^
B	26	6.00 ± 0.23 ^a^	5.04 ± 0.26 ^a^	1.15 ± 0.13 ^a^	4.88 ± 0.27 ^a^	3.50 ± 0.32 ^a^	1.08 ± 0.12 ^a^	4.73 ± 0.25 ^a^	3.08 ± 0.27 ^b^	4.85 ± 0.24 ^a^	1.08 ± 0.13 ^a^	4.96 ± 0.29 ^a^	5.92 ± 0.25 ^a^
C	26	6.04 ± 0.23 ^a^	4.81 ± 0.26 ^a^	1.27 ± 0.13 ^a^	4.31 ± 0.27 ^a,b^	3.35 ± 0.32 ^a^	1.23 ± 0.12 ^a^	4.35 ± 0.25 ^a^	3.12 ± 0.27 ^b^	4.69 ± 0.24 ^a^	1.15 ± 0.13 ^a^	5.35 ± 0.29 ^a^	6.03 ± 0.25 ^a^
D	26	6.15 ± 0.23 ^a^	4.81 ± 0.26 ^a^	1.23 ± 0.13 ^a^	4.04 ± 0.27 ^a,b^	3.46 ± 0.32 ^a^	1.23 ± 0.12 ^a^	3.69 ± 0.25 ^a,b^	3.73 ± 0.27 ^a,b^	3.92 ± 0.24 ^a,b^	1.31 ± 0.13 ^a^	5.54 ± 0.29 ^a^	5.42 ± 0.25 ^a,b^
E	26	6.12 ± 0.23 ^a^	4.19 ± 0.26 ^a^	1.27 ± 0.13 ^a^	3.50 ± 0.27 ^b^	3.62 ± 0.32 ^a^	1.27 ± 0.12 ^a^	3.12 ± 0.25 ^b^	4.12 ± 0.27 ^a,b^	3.54 ± 0.24 ^b^	1.35 ± 0.13 ^a^	5.58 ± 0.29 ^a^	4.65 ± 0.25 ^b^
F	26	5.46 ± 0.23 ^a^	5.04 ± 0.26 ^a^	1.23 ± 0.13 ^a^	4.88 ± 0.27 ^a^	3.38 ± 0.32 ^a^	1.15 ± 0.12 ^a^	4.38 ± 0.25 ^a^	3.46 ± 0.27 ^a,b^	4.54 ± 0.24 ^a,b^	1.15 ± 0.13 ^a^	5.19 ± 0.29 ^a^	5.73 ± 0.25 ^a,b^
G	26	5.62 ± 0.23 ^a^	4.85 ± 0.26 ^a^	1.35 ± 0.13 ^a^	4.23 ± 0.27 ^a,b^	3.50 ± 0.32 ^a^	1.46 ± 0.12 ^a^	3.81 ± 0.25 ^a,b^	3.73 ± 0.27 ^a,b^	4.12 ± 0.24 ^a,b^	1.50 ± 0.13 ^a^	5.62 ± 0.29 ^a^	5.42 ± 0.25 ^a,b^
H	26	5.54 ± 0.23 ^a^	4.96 ± 0.26 ^a^	1.27 ± 0.13 ^a^	4.85 ± 0.27 ^a^	3.65 ± 0.32 ^a^	1.15 ± 0.12 ^a^	4.77 ± 0.25 ^a^	3.50 ± 0.27 ^a,b^	4.81 ± 0.24 ^a^	1.31 ± 0.13 ^a^	6.04 ± 0.29 ^a^	5.50 ± 0.25 ^a,b^
I	26	5.73 ± 0.23 ^a^	5.15 ± 0.26 ^a^	1.23 ± 0.13 ^a^	4.54 ± 0.27 ^a,b^	3.54 ± 0.32 ^a^	1.62 ± 0.12 ^a^	4.27 ± 0.25 ^a^	3.85 ± 0.27 ^a,b^	4.27 ± 0.24 ^a,b^	1.42 ± 0.13 ^a^	5.81 ± 0.29 ^a^	5.08 ± 0.25 ^a,b^
Dataset average	221	5.83 ± 0.08	4.89 ± 0.09	1.25 ± 0.04	4.41 ± 0.10	3.54 ± 0.11	1.27 ± 0.04	4.04 ± 0.10	3.64 ± 0.09	4.30 ± 0.09	1.28 ± 0.04	5.49 ± 0.10	5.49 ± 0.09

Results are expressed as mean ± standard error. Values represented with different letters are statistically different at *p* ≤ 0.05; ^†^ significant factor in multifactor analysis; and ^‡^ not significant factor in multifactor analysis. A—control sample; 3DP fruit snacks with the addition of: B—saccharose, C—fructose, D—birch sugar (xylitol), E—erythritol, F— maple syrup, G—date syrup, H—agave syrup, I—stevia and erythritol; 1—lower level of sweeteners, 2—higher level of sweeteners.

## Data Availability

The data used to support the findings of this study can be made available by the corresponding author upon request.
